# Supplementary material for: Importance of ocean dynamics in the onset and persistence of the 2013-15 and 2019-20 northeast Pacific marine heatwaves
Source: Nat Commun. 2025 Nov 11;16:9935. doi: 10.1038/s41467-025-64873-2 (PMC12606263; doi:10.1038/s41467-025-64873-2)
Supplement: Supplementary file 1 — Supplementary Information [file 41467_2025_64873_MOESM1_ESM.pdf]

**SUPPLEMENTARY INFORMATION**

# Importance of ocean dynamics in the onset and persistence of the 2013-15 and 2019-20 northeast Pacific marine heatwaves

Yu Long et al

## Supplementary Note 1 | Introduction

Sea surface temperature anomalies (SSTA) encompass a range of signals, including interdecadal, interannual, interseasonal, and intraseasonal variations, as well as interactions across different time scales. To determine which specific signal leads to SSTA exceeding the defined threshold, one must analyze which signal exhibits a positive anomaly during the period of the temperature extreme and provide an explanation.

To streamline our analysis, we concentrate on the timescale that is most influential on the temperature extremes. The northeast Pacific is particularly noteworthy, as interannual climate variability appears to be the dominant cause of variations in northeast Pacific SSTA. Using the classic definition[1], we can identify the onset and end dates of marine heatwave events and calculate the average (or median) of both the unfiltered SSTA and filtered SSTA for different time scales (Figure SI-1).

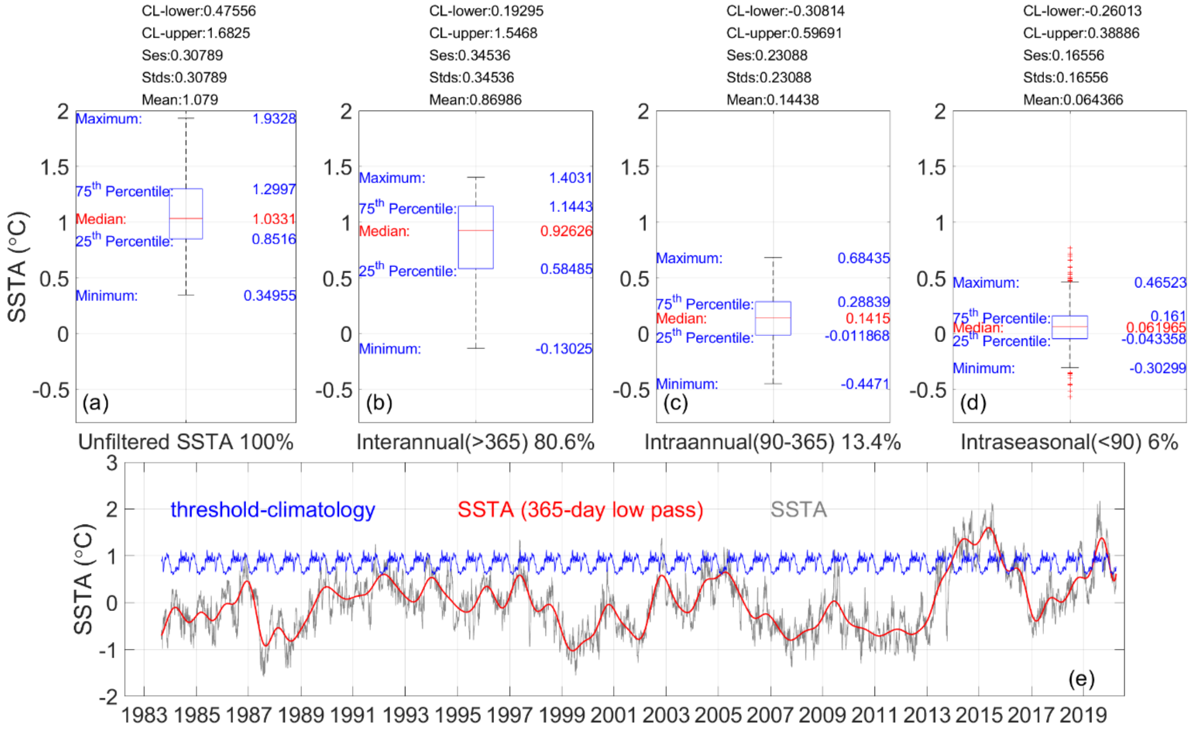


**Figure SI-1.** | Boxplot of (a) unfiltered SSTA, (b) its interannual component, (c) intra-annual component, and (d) intraseasonal component when the MHW occurred. The red line in the box denotes the median. (e) SSTA and threshold within 150 °W – 135 °W and 40 °N – 50 °N.

The interannual component contributes dominantly to temperature extremes during marine heatwave events, accounting for 80.7% of the mean value relative to the unfiltered SSTA ($\overline{SSTA_{Interannual-MHW}} \big/ \overline{SSTA_{unfiltered-MHW}}$). On the other hand, the interannual component of the mixed layer temperature anomalies (MLTA) accounted for 80% of the variations observed in the unfiltered MLTA ($STD(MLTA_{Interannual}) \big/ STD(MLTA_{unfiltered})$) (see Methods 5d). These substantial

proportions highlight the critical role of interannual-scale variability in driving extreme SSTA.

Alternatively, during the two ‘Blob’ events (i.e., the 2013-15 and 2019-20 events), the interannual component alone surpasses the threshold, demonstrating that understanding the interannual variability of SSTA is sufficient to explain the Blob dynamics. Therefore, we have applied the interannual time scale for our analysis.

## Supplementary Note 2 | Methods

The gridded temperature–salinity data from the Institute of Atmospheric Physics (IAP) are also employed to validate the meridional displacement of the gyre boundary. These data have a horizontal resolution of 1° and span 41 vertical levels from 1 to 2000 m[2]. Although the mean position of the gyre boundary derived from the IAP dataset is approximately 9° farther north than that from BRAN2020 (47.4° N compared to 38.4° N), and the gyre boundary varies between 46.4° N during the negative phase and 47.9° N during the positive phase, the variability of the gyre boundary in the IAP dataset exhibits a similar pattern to that in the BRAN2020 dataset.

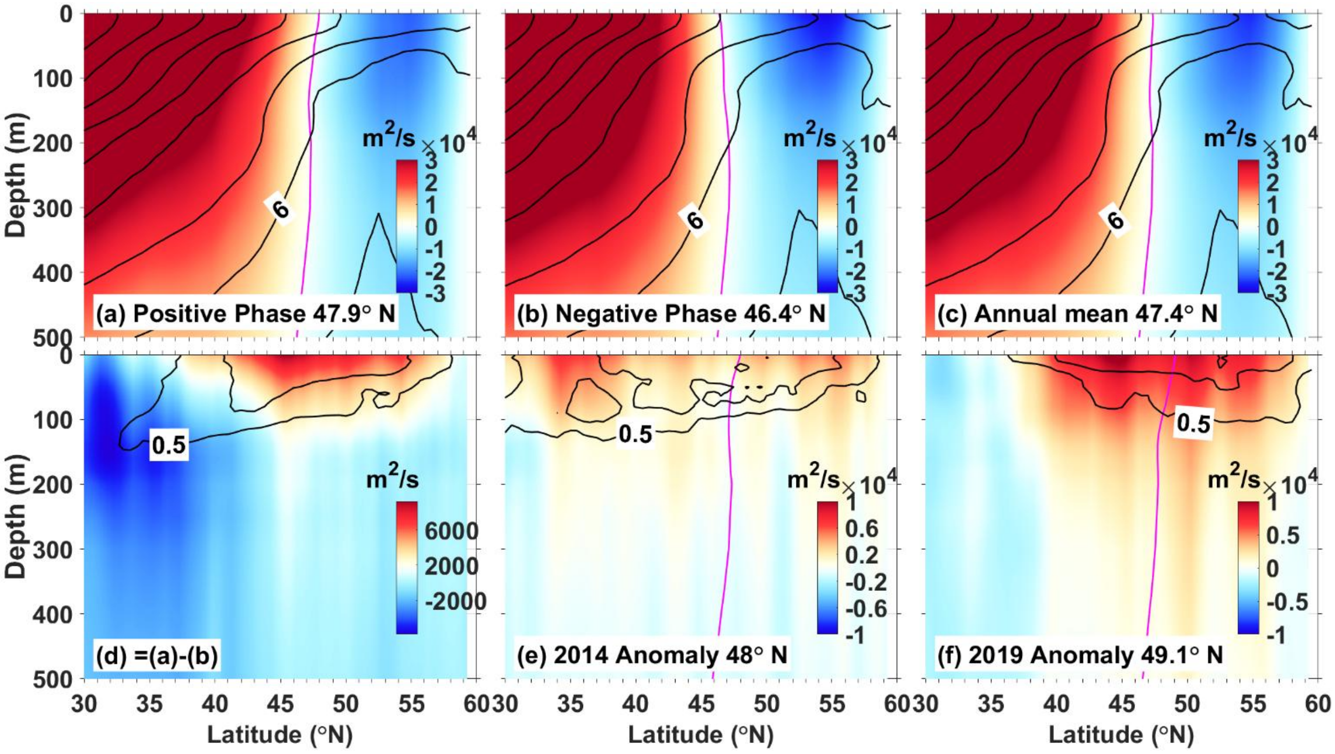


**Figure SI-2.** Same as Figure 5 but using IAP data. Zonally averaged (145 °W-135 °W) geostrophic stream function ($m^2/s$, color shading) and temperature (ºC, black line), relative to a reference depth of 2000 m, for the (a) positive phase, (b) negative phase, and (c) annual mean. (d) is the phase difference between (a) and (b). (e) and (f) are the anomaly for 2014 and 2019 from (c). The magenta line in (a, b, c, e, f) denotes the zero contour of the geostrophic stream function. Positive (negative) values of the geostrophic stream function in (d-f) represent anticyclonic (cyclonic) rotation (or change). The latitude in the legend is the position of the zero contour of the geostrophic stream function in the sea surface.

## Supplementary References


1. Hobday, A. J. et al. A hierarchical approach to defining marine heatwaves. Prog. Oceanogr. **141**, 227–238 (2016).

2. Cheng, L. et al. IAPv4 ocean temperature and ocean heat content gridded dataset. Earth System Science Data **16**, 3517–3546 (2024).
