# Supplementary material for: Importance of ocean dynamics in the onset and persistence of the 2013-15 and 2019-20 northeast Pacific marine heatwaves
Source: Nat Commun. 2025 Nov 11;16:9935. doi: 10.1038/s41467-025-64873-2 (PMC12606263; doi:10.1038/s41467-025-64873-2)
Supplement: Supplementary file 2 — Description of Additional Supplementary Files [file 41467_2025_64873_MOESM2_ESM.pdf]

Description of Additional Supplementary Files

File Name: Supplementary Data 1
Description: The data (in form of '.mat') and MATLAB code used to generate figure 1-6.

File Name: Supplementary Data 2
Description: The data (in form of '.mat') and MATLAB code used to generate figure 8.

File Name: Supplementary Data 3
Description: MATLAB toolboxes and functions that are called by the code in Supplementary Data 1 and Supplementary Data 2.
