# Supplementary figures and images for: Importance of ocean dynamics in the onset and persistence of the 2013-15 and 2019-20 northeast Pacific marine heatwaves
Source: Nat Commun. 2025 Nov 11;16:9935. doi: 10.1038/s41467-025-64873-2 (PMC12606263; doi:10.1038/s41467-025-64873-2)

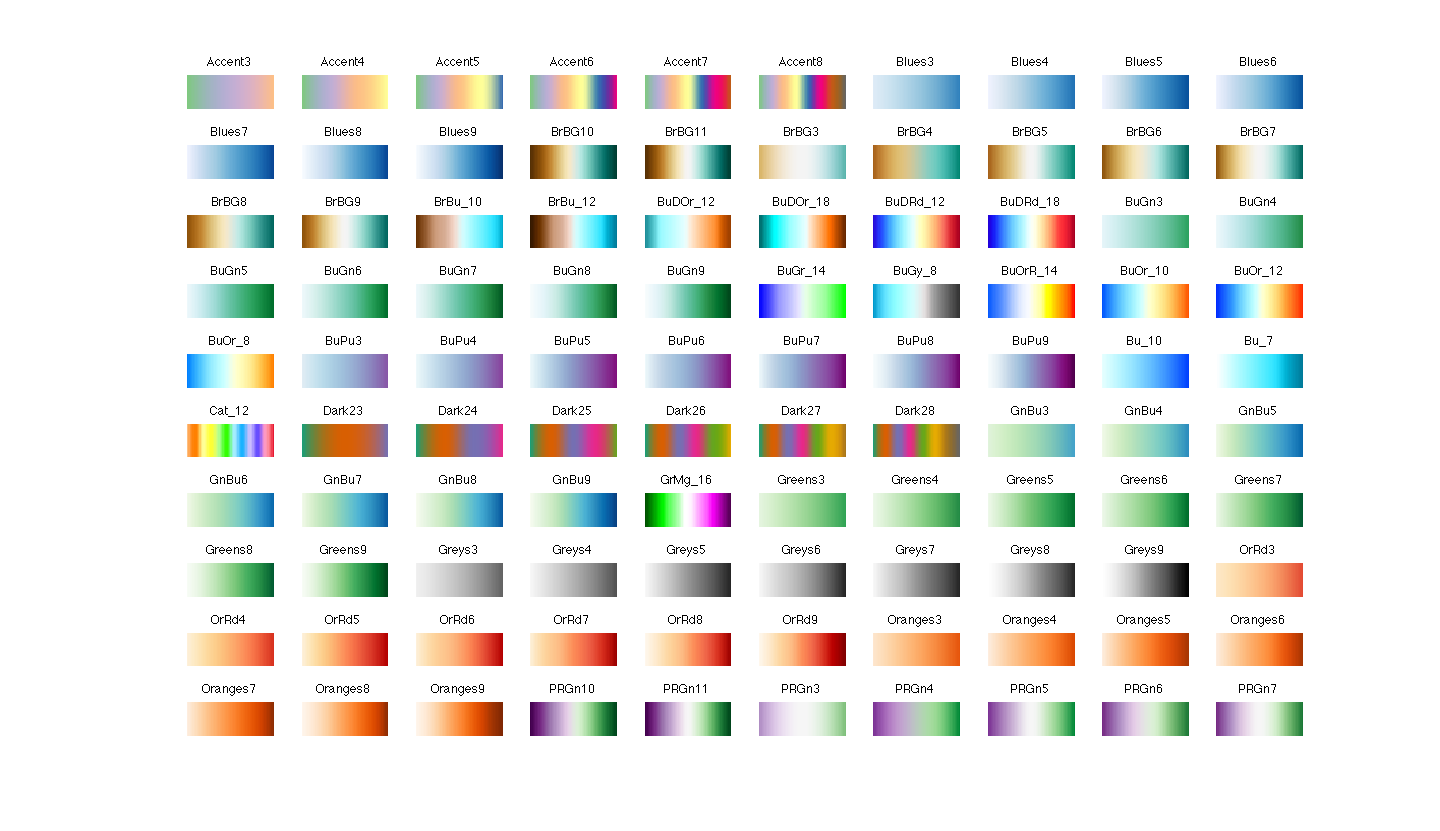

Supplement: Supplementary file 5 — Supplementary Data 3 [file 41467_2025_64873_MOESM5_ESM.zip › FunctionsOr_toolbox/othercolor/othercolor/othercolor1-100.png]

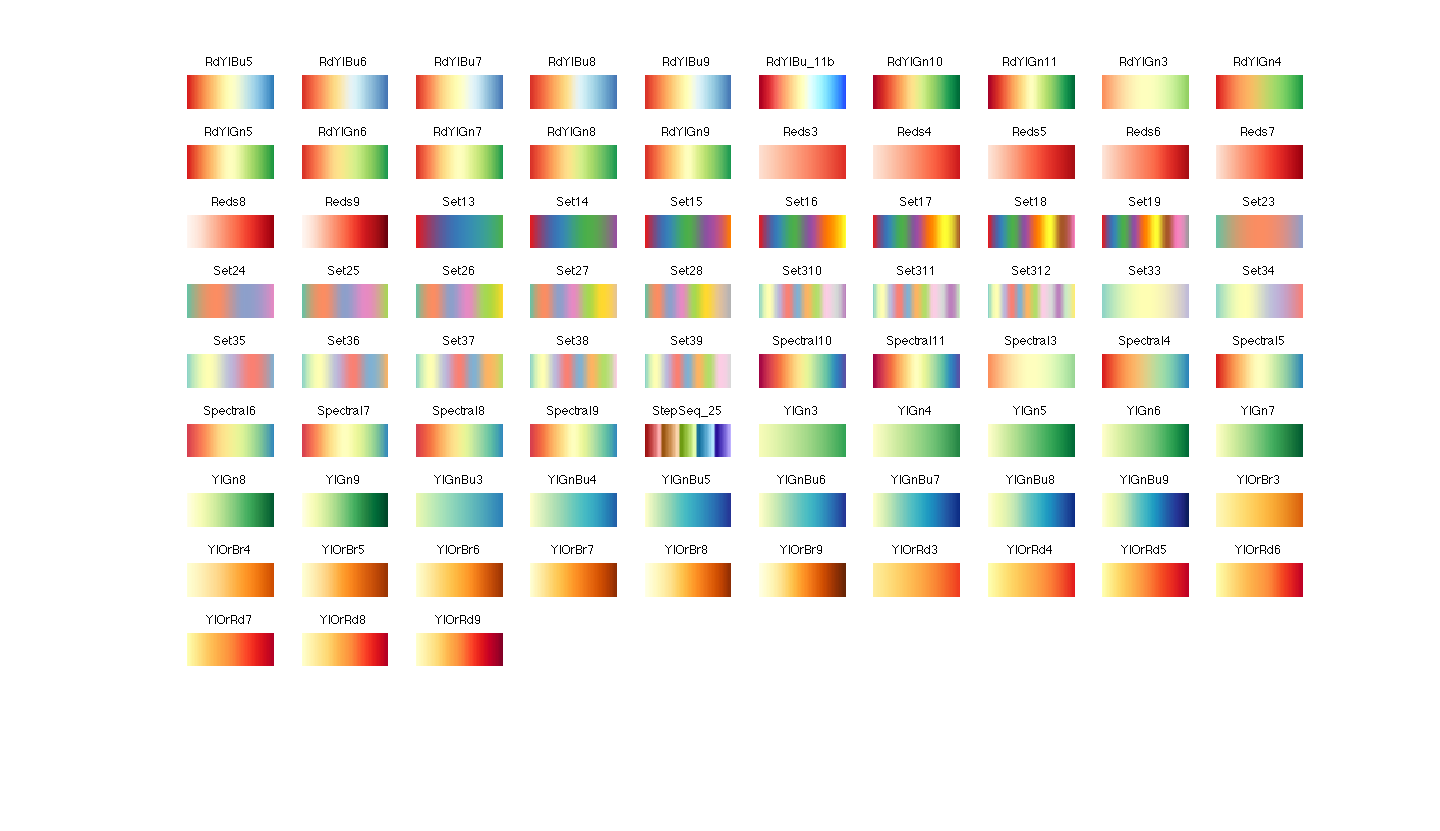

Supplement: Supplementary file 5 — Supplementary Data 3 [file 41467_2025_64873_MOESM5_ESM.zip › FunctionsOr_toolbox/othercolor/othercolor/othercolor201-283.png]

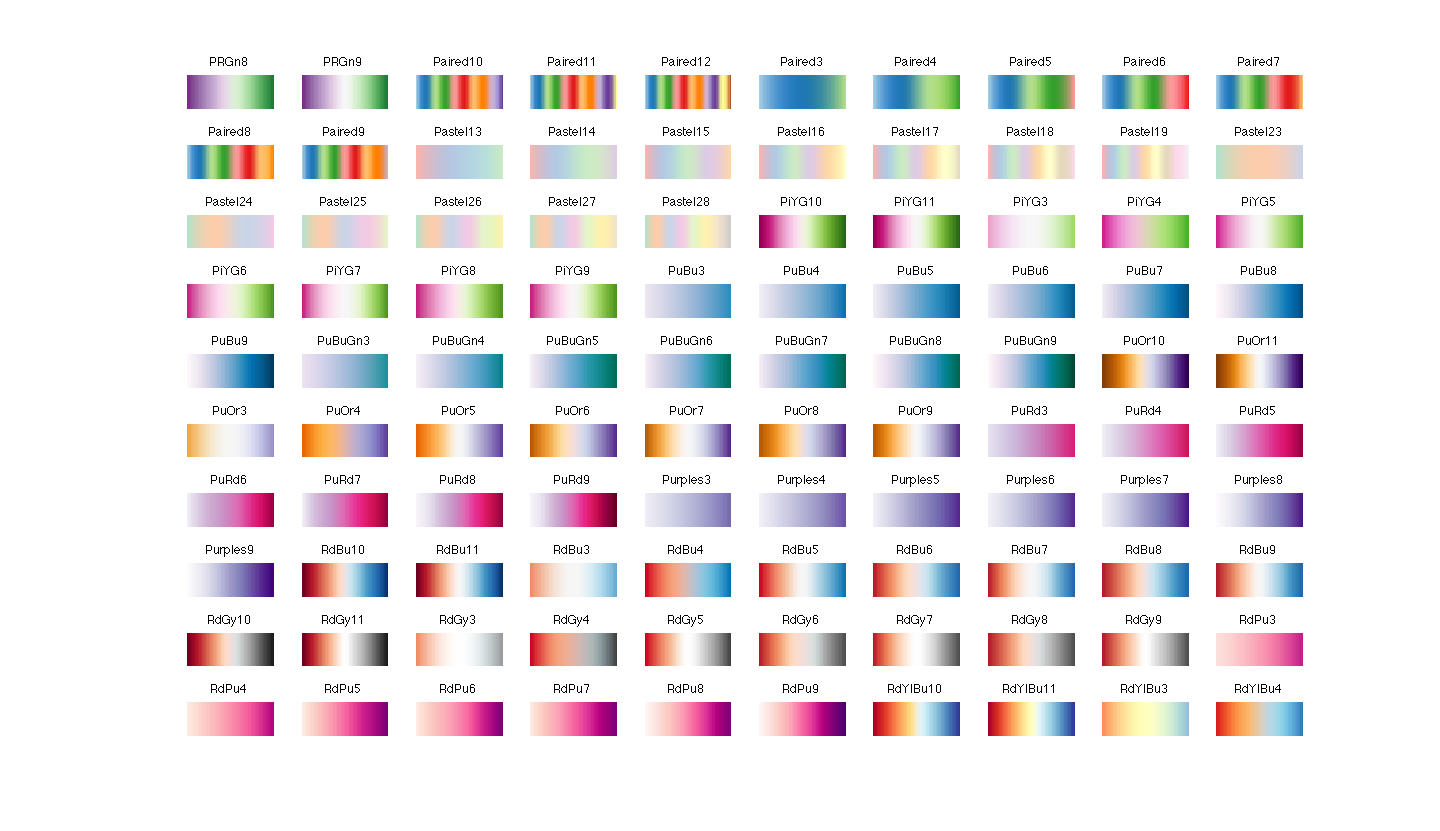

Supplement: Supplementary file 5 — Supplementary Data 3 [file 41467_2025_64873_MOESM5_ESM.zip › FunctionsOr_toolbox/othercolor/othercolor/othercolor_101-200.png]
